# Synergistic targeting of Sp1, a critical transcription factor for myeloma cell growth and survival, by panobinostat and proteasome inhibitors

**DOI:** 10.18632/oncotarget.12594

**Published:** 2016-10-12

**Authors:** Ariunzaya Bat-Erdene, Hirokazu Miki, Asuko Oda, Shingen Nakamura, Jumpei Teramachi, Ryota Amachi, Hirofumi Tenshin, Masahiro Hiasa, Masami Iwasa, Takeshi Harada, Shiro Fujii, Kimiko Sogabe, Kumiko Kagawa, Sumiko Yoshida, Itsuro Endo, Kenichi Aihara, Masahiro Abe

**Affiliations:** ^1^ Department of Hematology, Endocrinology and Metabolism, Institute of Biomedical Sciences, Tokushima University Graduate School of Medicine, Tokushima, Japan; ^2^ Division of Transfusion Medicine and Cell Therapy, Tokushima University Hospital, Tokushima, Japan; ^3^ Department of Orthodontics and Dentofacial Orthopedics, Tokushima University Graduate School of Oral Sciences, Tokushima, Japan; ^4^ Department of Histology and Oral Histology, Tokushima University Graduate School of Oral Sciences, Tokushima, Japan

**Keywords:** multiple myeloma, panobinostat, proteasome inhibitors, caspase-8, Sp1

## Abstract

Panobinostat, a pan-deacetylase inhibitor, synergistically elicits cytotoxic activity against myeloma (MM) cells in combination with the proteasome inhibitor bortezomib. Because precise mechanisms for panobinostat's anti-MM action still remain elusive, we aimed to clarify the mechanisms of anti-MM effects of panobinostat and its synergism with proteasome inhibitors. Although the transcription factor Sp1 was overexpressed in MM cells, the Sp1 inhibitor terameprocol induced MM cell death in parallel with reduction of IRF4 and cMyc. Panobinostat induced activation of caspase-8, which was inversely correlated with reduction of Sp1 protein levels in MM cells. The panobinostat-mediated effects were further potentiated to effectively induce MM cell death in combination with bortezomib or carfilzomib even at suboptimal concentrations as a single agent. Addition of the caspase-8 inhibitor z-IETD-FMK abolished the Sp1 reduction not only by panobinostat alone but also by its combination with bortezomib, suggesting caspase-8-mediated Sp1 degradation. The synergistic Sp1 reduction markedly suppressed Sp1-driven prosurvival factors, IRF4 and cMyc. Besides, the combinatory treatment reduced HDAC1, another Sp1 target, in MM cells, which may potentiate HDAC inhibition. Collectively, caspase-8-mediated post-translational Sp1 degradation appears to be among major mechanisms for synergistic anti-MM effects of panobinostat and proteasome inhibitors in combination.

## INTRODUCTION

Panobinostat is a novel pan-deacetylase inhibitor approved in many countries for use in combination with bortezomib and dexamethasone in relapsed or refractory patients with multiple myeloma (MM) [1]. Panobinostat has been demonstrated to synergistically elicit cytotoxic activity against MM cells in combination with bortezomib *in vitro* and *in vivo* [2, 3]. Inhibition of aggresome formation through the inhibition of HDAC6 by panobinostat and thereby potentiation of ER stress by bortezomib has been reported as a mechanism to contribute to this synergism [3, 4]. However, because panobinostat is able to widely inhibit histone deacetylase (HDAC) isoforms other than HDAC6, and because HDAC inhibitors have multiple mechanisms of action, including caspase-8 activation, there may be other mechanisms involved in the synergism between proteasome inhibitors and panobinostat.

Specificity protein 1 (Sp1) is a ubiquitous zinc-finger transcription factor that binds guanine–cytosine-rich elements in the promoter region of its target genes, and upregulates the expression of various important genes for cancer initiation and progression [5, 6]. Sp1 is known to be constitutively overexpressed in many cancers, and associated with poor prognosis [5]. In MM, Sp1 expression and its DNA binding activity have also been demonstrated to be upregulated; inhibition of Sp1 expression using Sp1 siRNA markedly suppressed MM cell growth and induced apoptosis, suggesting Sp1 as a novel therapeutic target for MM [7].

Sp1 protein expression and its transcriptional activity are highly regulated by post-translational modifications [5]. The reduction of Sp1 protein levels has been demonstrated to be induced in MM cells by bortezomib largely through caspase-8 activation and thereby enzymatic Sp1 protein degradation, indicating a predominant role of caspase-8 activation in post-translational Sp1 protein degradation [8, 9]. Because panobinostat has multiple proposed mechanisms of action, and because anti-MM effects of panobinostat still remains to be clarified, in the present study we aimed to clarify the mechanisms of anti-MM effects of panobinostat and its synergism with proteasome inhibitors, focusing on degradation of the transcription factor Sp1. We demonstrate here that Sp1 is overexpressed in MM cells to act as a critical mediator for MM cell growth and survival, and that bortezomib or carfilzomib enhanced caspase-8-mediated Sp1 degradation to effectively induce MM cell death in combination with panobinostat. The synergistic Sp1 reduction markedly suppressed Sp1-driven prosurvival factors, interferon regulatory factor 4 (IRF4) and cMyc, while potentiating HDAC inhibition in part through HDAC1 reduction in MM cells. Therefore, caspase-8-mediated post-translational Sp1 degradation appears to be among major mechanisms for synergistic anti-MM effects of panobinostat and proteasome inhibitors in combination.

## RESULTS

### Sp1 inhibition induces MM cell death

**Figure 1:**
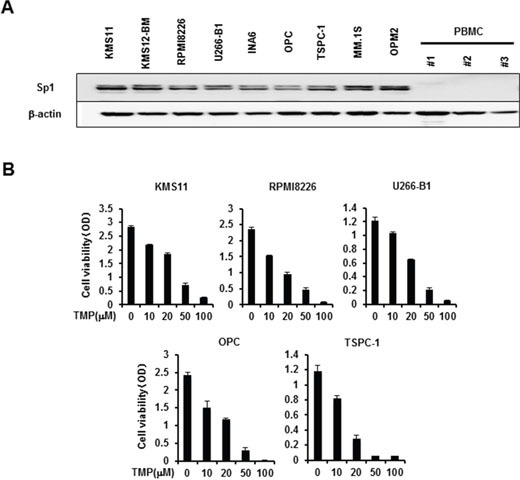
Sp1 expression in MM cells and MM cell viability by Sp1 inhibition **A.** Cell lysates were extracted from MM cell lines as indicated and peripheral blood mononuclear cells (PBMC) isolated from 3 normal donors. The protein levels of Sp1 were analyzed by Western blotting. β-actin was used as a protein loading control. **B**. The indicated MM cell lines were cultured in triplicate in the absence or presence of the Sp1 inhibitor terameprocol (TMP) at the indicated concentrations. After culturing for 48 hours, cell viability was measured by a WST-8 cell proliferation assay. Results were expressed as the mean +/− SD.

We first examine the expression of Sp1 protein in MM cells. Consistent with the previous report [7], Sp1 protein was overexpressed in all MM cell lines tested, whereas only marginally expressed in peripheral blood mononuclear cells from normal subjects (Figure 1A). To clarify the role of Sp1 in MM cell growth and survival, we next examined the effects of the Sp1 inhibitor terameprocol (TMP), which competitively inhibits Sp1 binding to DNA. Treatment with TMP dose-dependently suppressed MM cell viability (Figure 1B). These results suggest therapeutic potential of targeting Sp1 up-regulated in MM cells.

### Panobinostat induces caspase-8-dependent Sp1 protein degradation in MM cells

**Figure 2:**
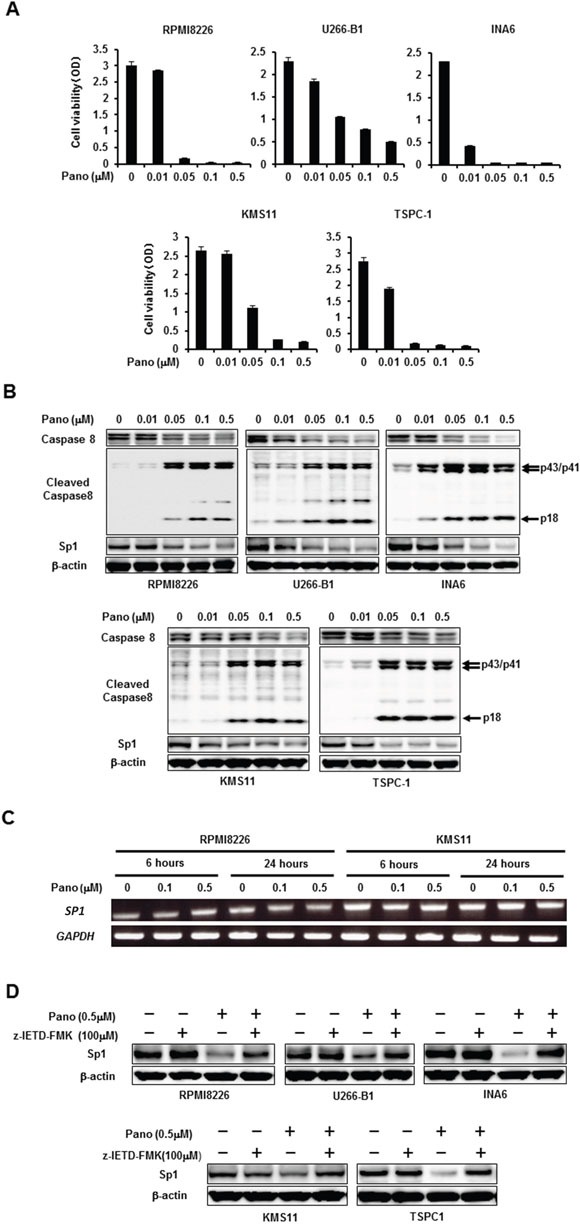
Post-translational reduction of Sp1 in MM cells by panobinostat **A.** The indicated MM cell lines were cultured in triplicate in the absence or presence of panobinostat at the indicated concentrations. After culturing for 48 hours, cell viability was measured by a WST-8 cell proliferation assay. Results were expressed as the mean +/− SD. **B.** The indicated MM cell lines were cultured for 24 hours in the absence or presence of panobinostat at the indicated concentrations. Then, the protein levels of caspase-8, cleaved caspase 8 and Sp1 were analyzed by Western blotting. **C.** RPMI8226 and KMS11 cells were cultured in the absence or presence of panobinostat at the indicated concentrations for 6 and 24 hours, and *SP1* mRNA expression was analyzed by RT-PCR. *GAPDH* was used as an internal control. **D.** The indicated MM cell lines were cultured for 24 hours in the absence or presence of panobinostat at 0.5 μM. The caspase-8 inhibitor z-IETD-FMK was added at 100 μM as indicated. Then, the protein levels of Sp1 were analyzed by Western blotting. β-actin was used as a protein loading control. Pano, panobinostat.

Because HDAC inhibitors are known to preferentially induce caspase-8 activation to contribute to tumor cell death [10], we asked whether panobinostat induces caspase-8-dependent Sp1 protein degradation in MM cells. Treatment with panobinostat reduced viability of all MM cell lines in a dose-dependent manner (Figure 2A). In parallel with the MM cell death, panobinostat dose-dependently induced activation of caspase-8 as determined by induction of cleaved caspase-8 fragments, which inversely correlated with reduction of Sp1 protein levels in the MM cells (Figure 2B). However, panobinostat up to 0.5 μM, the concentration enough to maximally diminish Sp1 protein levels in the MM cells, did not affect the *SP1* mRNA levels (Figure 2C), suggesting a post-translational mechanism of the Sp1 reduction by panobinostat. To clarify the role of the caspase-8 activation in the Sp1 reduction, we next investigated the effects of the caspase-8 inhibitor z-IETD-FMK on Sp1 protein levels in MM cells in the absence or presence of 0.5 μM panobinostat. Addition of z-IETD-FMK mostly inhibited the panobinostat-induced down-regulation of Sp1 reduction in all MM cell lines tested (Figure 2D), indicating a predominant role of caspase-8-mediated Sp1 protein degradation by panobinostat.

### Cooperative Sp1 protein reduction and MM cell death by panobinostat and proteasome inhibitors in combination

**Figure 3:**
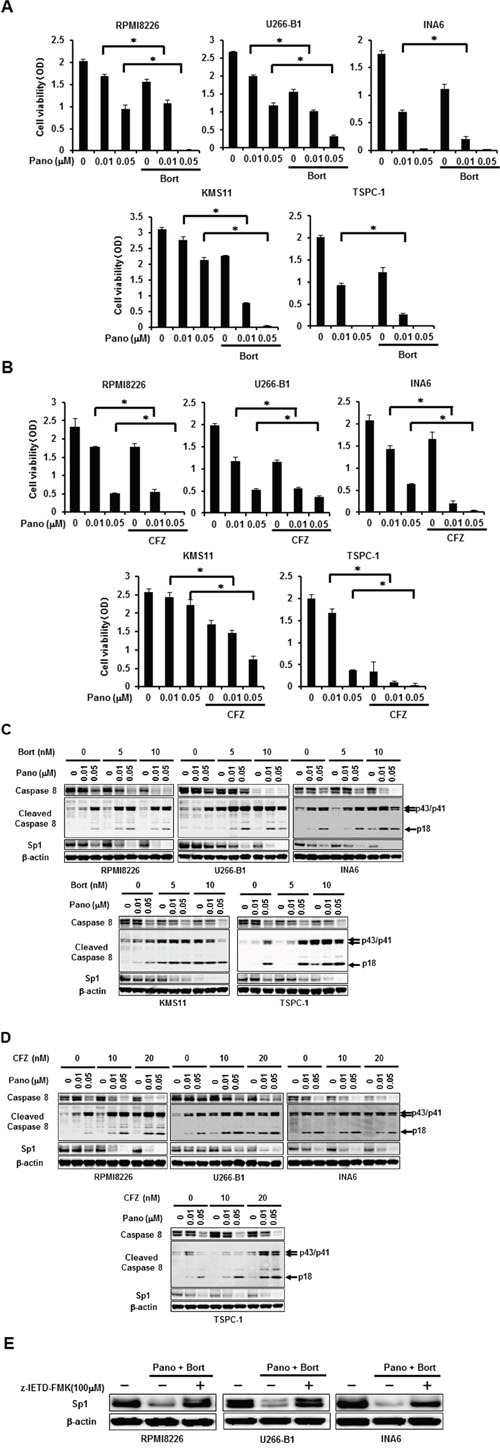
Synergistic reduction of MM cell viability and Sp1 protein levels by panobinostat and proteasome inhibitors **A, B.** The indicated MM cell lines were cultured in triplicate in the absence or presence of panobinostat at the indicated concentrations. Bortezomib was added at 5 nM (A) or carfilzomib was added at 20 nM (B) as indicated. After culturing for 48 hours, cell viability was measured. Results were expressed as the mean +/− SD. *p, <0.05. **C, D.** The indicated MM cell lines were cultured for 24 hours in the absence or presence of panobinostat at the indicated concentrations. Bortezomib (C) or carfilzomib (D) was concomitantly added as indicated. Then, the protein levels of caspase-8, cleaved caspase 8 and Sp1 were analyzed by Western blotting. **E.** RPMI8226, U266-B1 and INA6 cells were cultured for 24 hours in the absence or presence of panobinostat at 0.05 μM and bortezomib at 5 nM in combination. The caspase-8 inhibitor z-IETD-FMK was added at 100μM as indicated. Then, the protein levels of Sp1 were analyzed by Western blotting. β-actin was used as a protein loading control. Pano, panobinostat; Bort, bortezomib; CFZ, carfilzomib.

According to the previous observation with the caspase-8 activation-mediated Sp1 protein degradation by bortezomib [8], additive effects of panobinostat with proteasome inhibitors can be expected in terms of Sp1 protein degradation by caspase-8. Therefore, we next examined the combinatory effects of panobinostat plus proteasome inhibitors on viability and Sp1 levels in MM cells. Treatment with bortezomib at 5 nM (Figure 3A) or carfilzomib at 20 nM (Figure 3B) alone only partially induced cell death in MM cells; however, bortezomib or carfilzomib at these suboptimal concentrations enhanced MM cell death in combination with panobinostat even at 0.01 μM, the suboptimal concentration for MM cell death induction as a single agent, indicating cooperative induction of MM cell death by panobinostat and these proteasome inhibitors. We next looked at Sp1 protein levels in MM cells upon these combinatory treatments. Panobinostat dose-dependently induced activation of caspase-8 and reduced Sp1 protein levels, which was further potentiated by addition of bortezomib (Figure 3C) or carfilzomib (Figure 3D). Similar to MM cell lines, panobinostat and bortezomib cooperatively activated caspase-8 with Sp1 reduction and induced cell death in primary MM cells (Supplementary Figure S1). To determine the role of the caspase-8 activation in Sp1 protein downregulation, we examined the effects of the caspase-8 inhibitor z-IETD-FMK on Sp1 protein levels in MM cells in the absence or presence of panobinostat at 0.05 μM and bortezomib at 5 nM in combination. Addition of z-IETD-FMK antagonized the Sp1 reduction by the combination of panobinostat and bortezomib and maintained high Sp1 protein levels in MM cells (Figure 3E), confirming the predominant role of caspase-8 activation in synergistic downregulation of Sp1 protein by panobinostat plus bortezomib. These results suggest synergistic cytotoxic effects with Sp1 reduction of panobinostat and these proteasome inhibitors at suboptimal concentrations as a single agent.

### Panobinostat and proteasome inhibitors cooperatively reduce cMyc and IRF4

**Figure 4:**
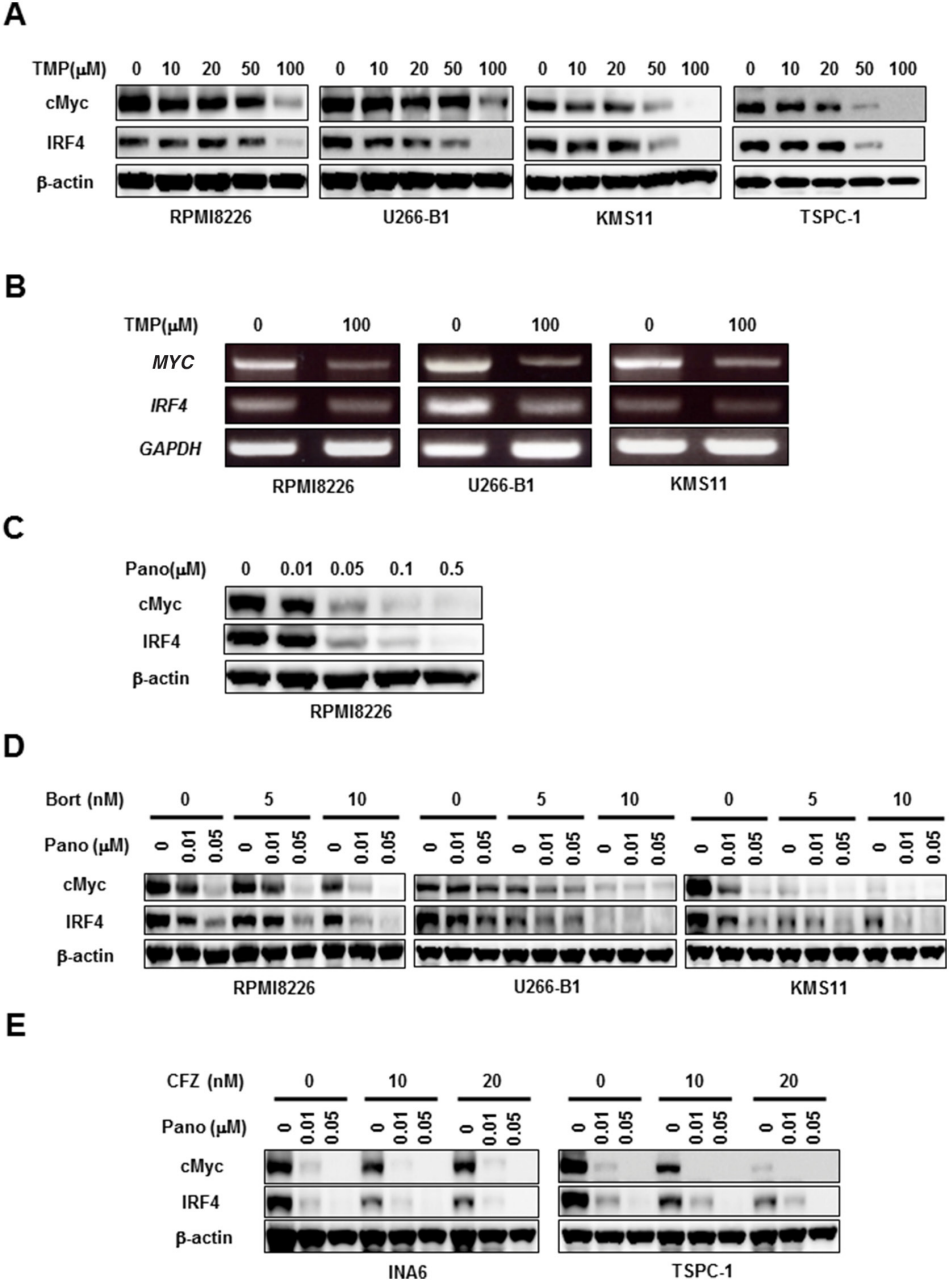
Panobinostat and proteasome inhibitors cooperatively reduce cMyc and IRF4 **A, B.** The indicated MM cell lines were cultured for 24 hours in the absence or presence of terameprocol at the indicated concentrations. Then, the protein levels of cMyc and IRF4 were analyzed by Western blotting (A). *MYC* and *IRF4* mRNA expression was analyzed by RT-PCR (B). *GAPDH* was used as an internal control. **C.** RPMI8226 cells were cultured for 24 hours in the absence or presence of panobinostat at the indicated concentrations. Then, the protein levels of cMyc and IRF4 were analyzed by Western blotting. **D, E.** The indicated MM cell lines were cultured for 24 hours in the absence or presence of panobinostat at the indicated concentrations. Bortezomib (D) or carfilzomib (E) was concomitantly added as indicated. Then, the protein levels of cMyc and IRF4 were analyzed by Western blotting. β-actin was used as a protein loading control. TMP, terameprocol; Pano, panobinostat; Bort, bortezomib; CFZ, carfilzomib.

cMyc and IRF4 have drawn considerable attention as critical pro-survival factors for MM cells [11, 12]. Interestingly, treatment with TMP dose-dependently reduced cMyc and IRF4 at protein as well as mRNA levels (Figures 4A and 4B, respectively), indicating the regulatory role of Sp1 in cMyc and IRF4 expression in MM cells. As expected from the observation that Sp1 was reduced in MM cells by panobinostat (Figure 2B), panobinostat dose-dependently decreased cMyc and IRF4 in MM cells (Figure 4C). Consistent with synergistic Sp1 reduction by panobinostat and the proteasome inhibitors, bortezomib (Figure 4D) or carfilzomib (Figure 4E) in combination with panobinostat further diminished cMyc and IRF4 levels in MM cells. These results suggest that the combinatory treatment of panobinostat and proteasome inhibitors efficaciously reduces the protein levels of Sp1 target molecules, including cMyc and IRF4, which may at least in part contribute to MM cell death.

### Panobinostat and proteasome inhibitors cooperatively enhance HDAC inhibition in MM cells

**Figure 5:**
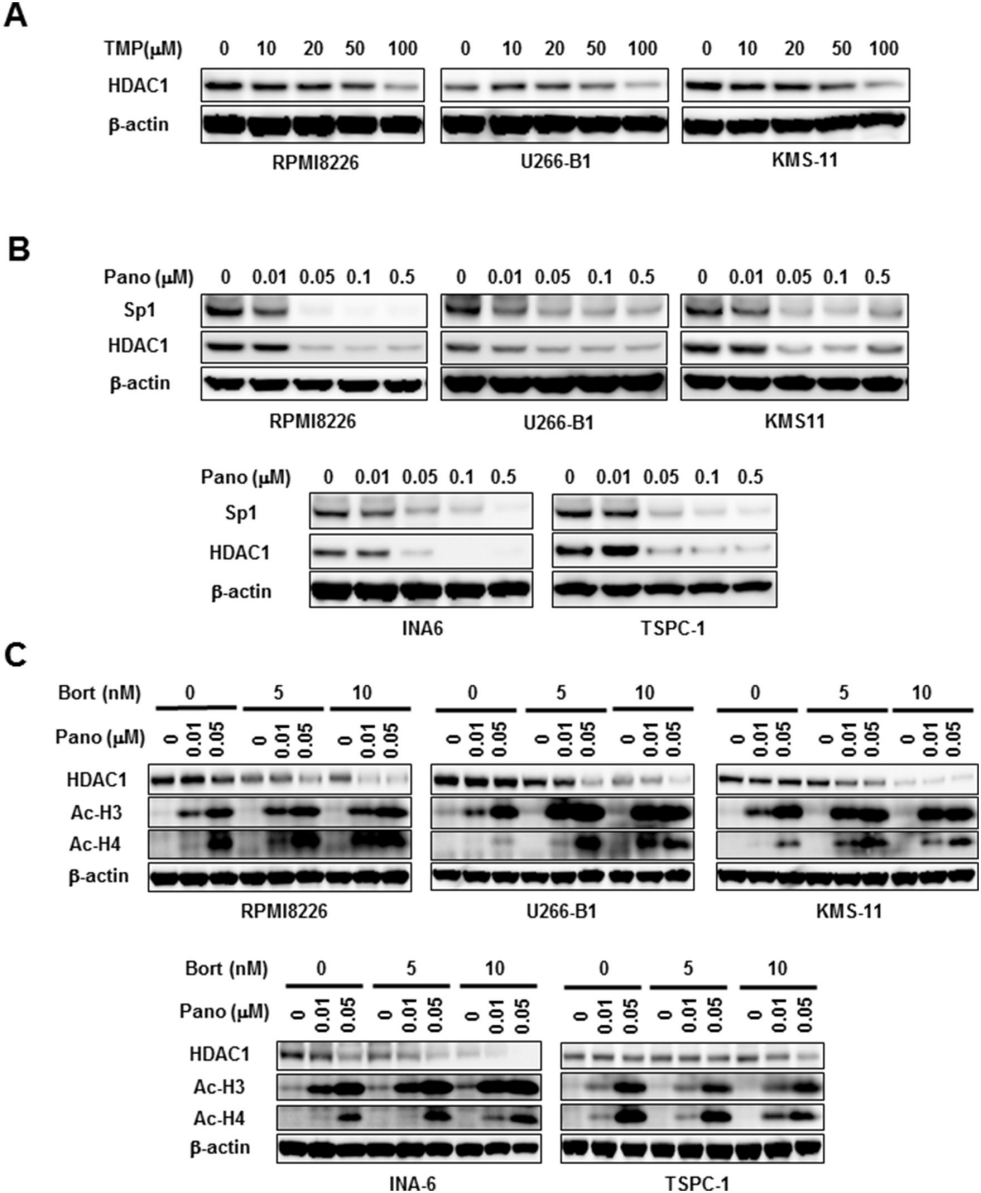
HDAC1 reduction by panobinostat and bortezomib in combination **A.** The indicated MM cell lines were cultured for 24 hours in the absence or presence of TMP at the indicated concentrations. Then, the protein levels of Sp1 were analyzed by Western blotting. **B.** The indicated MM cell lines were cultured for 24 hours in the absence or presence of panobinostat at the indicated concentrations. Then, the protein levels of Sp1 and HDAC1 were analyzed by Western blotting. **C.** The indicated MM cell lines were cultured for 24 hours in the absence or presence of panobinostat at the indicated concentrations. Bortezomib was added as indicated. Then, the protein levels of HDAC1 and acetylated histone H3 (Ac-H3) and acetylated histone H4 (Ac-H4) were analyzed by Western blotting. β-actin was used as a protein loading control. TMP, terameprocol; Pano, panobinostat; Bort, bortezomib.

Sp1 is regarded as a potent transactivator of class1 HDAC genes, including the *HDAC1* gene [8, 9]. The Sp1 inhibitor TMP dose-dependently curtailed HDAC1 protein levels in MM cells (Figure 5A), confirming induction of HDAC1 expression by Sp1 in MM cells. Consistently, panobinostat dose-dependently decreased HDAC1 levels in MM cells in parallel with Sp1 reduction (Figure 5B). Because panobinostat plus bortezomib or carfilzomib synergistically induced caspase-8-mediated post-translational Sp1 degradation in MM cells, we next looked at the effects of bortezomib in combination with panobinostat on HDAC1 expression in MM cells. Bortezomib further reduced HDAC1 levels in MM cells in the presence of panobinostat (Figure 5C), indicating cooperative suppression of HDAC1 expression. Finally, we examined the status of histone acetylation in MM cells upon the combinatory treatment with panobinostat plus bortezomib. Panobinostat dose-dependently induced the acetylation of histone H3 and histone H4 in MM cells, which was further potentiated by addition of bortezomib (Figure 5C). These results suggest that the combinatory treatment may lead to further potentiation of HDAC inhibition at least in part through HDAC1 reduction.

## DISCUSSION

The present study demonstrates that Sp1 is overexpressed in MM cells to act as a critical mediator for MM cell growth and survival, and that combinatory treatment with panobinostat and proteasome inhibitors efficaciously degrades Sp1 through caspase-8 activation to cause MM cell death.

IRF4 is regarded as an ‘achilles heel’ of MM cells, and regulates the expression of a wide variety of genes associated with MM cell growth and survival, indicating non-oncogenic addiction of MM cells to IRF4 [12]. Furthermore, IRF4 and cMyc have been demonstrated to interact with each other to enhance their expression in MM cells [12, 13]. IRF4 and cMyc have drawn considerable attention as critical pro-survival factors for MM cells [11, 12], especially after unveiling the mechanisms of action for anti-MM effects of immunomodulatory agents (IMiDs), including lenalidomide and pomalidomide. IMiDs have been demonstrated to bind to cereblon to trigger proteasomal degradation of the transcription factors Ikaros (IKZF1) and Aiolos (IKZF3) [14, 15], which results in reduction of IRF4 and cMyc and thereby MM cell death [16, 17]. Thus, IRF4 and cMyc are now generally accepted as critical prosurvival mediators to be targeted in MM cells. The present study demonstrates that IRF4 and cMyc expression is driven by Sp1 overexpressed in MM cells, and that Sp1 degradation by combinatory treatment with panobinostat and proteasome inhibitors causes substantial reduction of IRF4 and cMyc, which at least in part contributes to the induction of MM cell death.

The combinatory treatment with panobinostat and proteasome inhibitors also curtails protein levels in MM cells of HDAC1, another target molecule of Sp1, which may lead to further potentiation and prolongation of HDAC inhibition in cooperation with direct immediate enzymatic HDAC inhibition by panobinostat. HDAC isoform levels have been reported to be dysregulated in MM cells, and higher HDAC1 levels in MM cells correlated with poor prognosis in patients with MM [18]. The synergistic suppression of HDAC1 expression by panobinostat and proteasome inhibitors may affect the epigenetic nature of MM cell pathogenesis.

The mechanism of activation of caspase-8 in MM cells by panobinostat still remains largely unknown. HDAC inhibition by valproic acid has been demonstrated to upregulate TRAIL and its receptor DR5 in acute myeloid leukemia cells and thereby induces caspase-8 activation [19]. Similar to the effects of valproic acid on acute myeloid leukemia cells as reported, treatment with panobinostat upregulated the expression of TRAIL and DR5 on the surface of MM cells (manuscript in preparation). Therefore, such MM cells may interact with each other to induce caspase-8 activation. In addition, the treatment with panobinostat reduced the expression of cFLIP, an endogenous inhibitor of caspase-8, in MM cells, which is able to further potentiate the activation of caspase-8 (manuscript in preparation). Because the Sp1 inhibitor terameprocol dose-dependently reduced cFLIP expression in MM cells (data not shown), caspase-8-mediated degradation of Sp1 can be involved in the reduction of cFLIP expression in MM cells by panobinostat.

Our study also demonstrates that Sp1 is a *bona fide* target of caspase-8 in MM cells. Novel therapeutic strategies targeting Sp1 can be envisioned by combination of panobinostat with other potent inducers of caspase-8 activation such as TNF-related apoptosis-inducing ligand agonists. Furthermore, because a growing body of evidence indicates a critical role of Sp1 in tumor initiation and/or maintenance of self-renewal capacity in various types of cancers [5], Sp1 inhibition with these strategies may impair MM initiating cells or progenitors together with MM cells with a mature phenotype.

Collectively, caspase-8-mediated post-translational Sp1 degradation appears to be among major mechanisms of the synergistic anti-MM effects by panobinostat and proteasome inhibitors in combination, aside from the induction of direct caspase-8 and caspase-9-dependent apoptosis [20, 21], and potentiation of ER stress [3]. Suppression of aggresome formation by HDAC6 inhibition to strengthen ER stress and degradation of Sp-1 mediated by caspase-8 activation are thought to be induced in parallel in MM cells by panobinostat and proteasome inhibitors in combination. However, the expression levels of Sp1 or HDAC6 may affect the induction of these mechanisms in MM cells. Or, these mechanisms may be differently induced when MM cells acquire the resistance to ER stress or caspase-8 activation.

## MATERIALS AND METHODS

### Reagents

The following reagents purchased from the indicated manufacturers: rabbit polyclonal anti-human Sp1, IRF4, cleaved caspase-8 antibody, mouse polyclonal anti-human caspase-8 antibody, horseradish peroxidase (HRP)-anti-rabbit IgG, anti-mouse IgG and bortezomib from Cell Signaling Technology (Beverly, MA); rabbit polyclonal anti-human cMyc antibody and anti-HDAC1 antibody from Abcam (Cambridge, UK); anti-acetylated histone H3, acetylated histone H4 (Merk Millipore, Billerica, MA); mouse monoclonal anti-β-actin antibody and terameprocol from Sigma-Aldrich (St. Louis, MO); panobinostat from Cayman Chemical Company (Ann Arbor, MI); Z-lle-Glu(O-ME)-Thr-Asp(O-Me) fluoromethyl ketone (Z-IETD-FMK) from TONBO biosciences (San Diego, CA) and carfilzomib from Chemie Tek (Indianapolis, Indiana) respectively.

### Cells and cell culture

The use of human samples was approved by the Institutional Review Board at Tokushima University, and informed consent was obtained according to the Declaration of Helsinki. Peripheral blood mononuclear cells were isolated from fresh peripheral blood as previously described [22]. The human MM cell lines RPMI-8226, U266-B1 and KMS-11 were obtained from the American Type Culture Collection (ATCC, Rockville, MD); TSPC-1 and OPC were established in our laboratory [23]. The human MM cell lines INA-6 were kindly provided by Dr. Renate Burger (University of Kiel, Kiel, Germany). Cells were cultured in RPMI1640 medium (Sigma, Aldrich, MO) supplemented with 10% FBS (Life Technologies, Grand Island, NY), penicillin G at 50 μg/mL and streptomycin at 50 μg/mL.

### Reverse transcription-polymerase chain reaction (RT-PCR)

Total RNA was extracted using TRIZOL reagent (Gibco BRL, Rockville, MD). Two μg total RNA was reverse-transcribed with Superscript II (Gibco) in a 20 μL reaction solution. One tenth of the RT-PCR products were used for subsequent PCR analysis with 23–30 cycles of 95°C for 30 seconds, 58°C for 30 seconds, and 72°C for 30 seconds. The primers used were listed in (Table 1).

**Table 1:** The list of primers

	Sense	Antisense
*MYC*	5′- TTCCCCTACCCTCTCAACGACAG -3′	5′- TCCTTACTTTTCCTTACGCACAA -3′
*IRF4*	5′- TTAATTCTCCAAGCGGATGC -3′	5′- AAGGAATGAGGAAGCCGTTC -3′
*SP1*	5′- TTGAAAAAGGAGTTGGTGGC -3′	5′- TGCTGGTTCTGTAAGTTGGG -3′
*GAPDH*	5′- TGTCTTCACCACCATGGAGAAGG -3′	5′- GTGGATGCAGGGATGATGTTCTG -3′

### Cell viability

Cell viability was determined by Cell Counting Kit-8 assay (Dojindo, Kumamoto, Japan) according to the manufacturer's instructions. Briefly, cells were plated in a 96-well plate and incubated with 2-(2-methoxy-4-nitrophenyl)-3-(4-nitrophenyl)-5-(2,4-disulphophenyl)-2H-tetrazolium monosodium salt (WST-8). After the incubation, the absorbance of each well was measured at 450-655 nm with iMark™ microplate reader (Bio-Rad Laboratories, Hercules, CA).

### Western blot analysis

Cells were collected and lysed in lysis buffer (Cell Signaling, Beverly, MA) supplemented with 1 mmoL/L phenylmethylsulfonyl fluoride and protease inhibitor cocktail solution (Sigma-Aldrich). The cell lysates were separated by sodium dodecyl sulfate polyacrylamide gel electrophoresis (SDS-PAGE) on a 10% polyacrylamide gel, and transferred to polyvinylidene difluoride membranes (Millpore, Billerica, MA). Membranes were blocked with 3% non-fat dry milk in Tris-buffered saline with 0.01% Tween 20 for 1 hour at room temperature and incubated for 16 hour at 4°C with primary antibodies. After washing, secondary horseradish peroxidase-conjugated antibody was added, and membranes were then developed, using the enhanced chemiluminescence plus Western blotting detection system (American Biosciences, Piscataway, NJ).

### Statistical analysis

Statistical analysis was performed using Student's *t*-test or one-way analysis of variance (ANOVA). P ≤ 0.05 was considered as a significant difference. All statistics were performed using the Statistical Package for Social Sciences (SPSS 13.0 for Windows; Chicago, IL).

## SUPPLEMENTARY FIGURES


