# Supplementary material for: Synergistic targeting of Sp1, a critical transcription factor for myeloma cell growth and survival, by panobinostat and proteasome inhibitors
Source: Oncotarget. 2016 Oct 12;7(48):79064–75. doi: 10.18632/oncotarget.12594 (PMC5346698; doi:10.18632/oncotarget.12594)
Supplement: Supplementary file 1 [file oncotarget-07-79064-s001.pdf]

Synergistic targeting of transcription factor specificity protein 1, a critical transcription factor for myeloma cell growth and survival, by panobinostat and proteasome inhibitors

SUPPLEMENTARY FIGURE

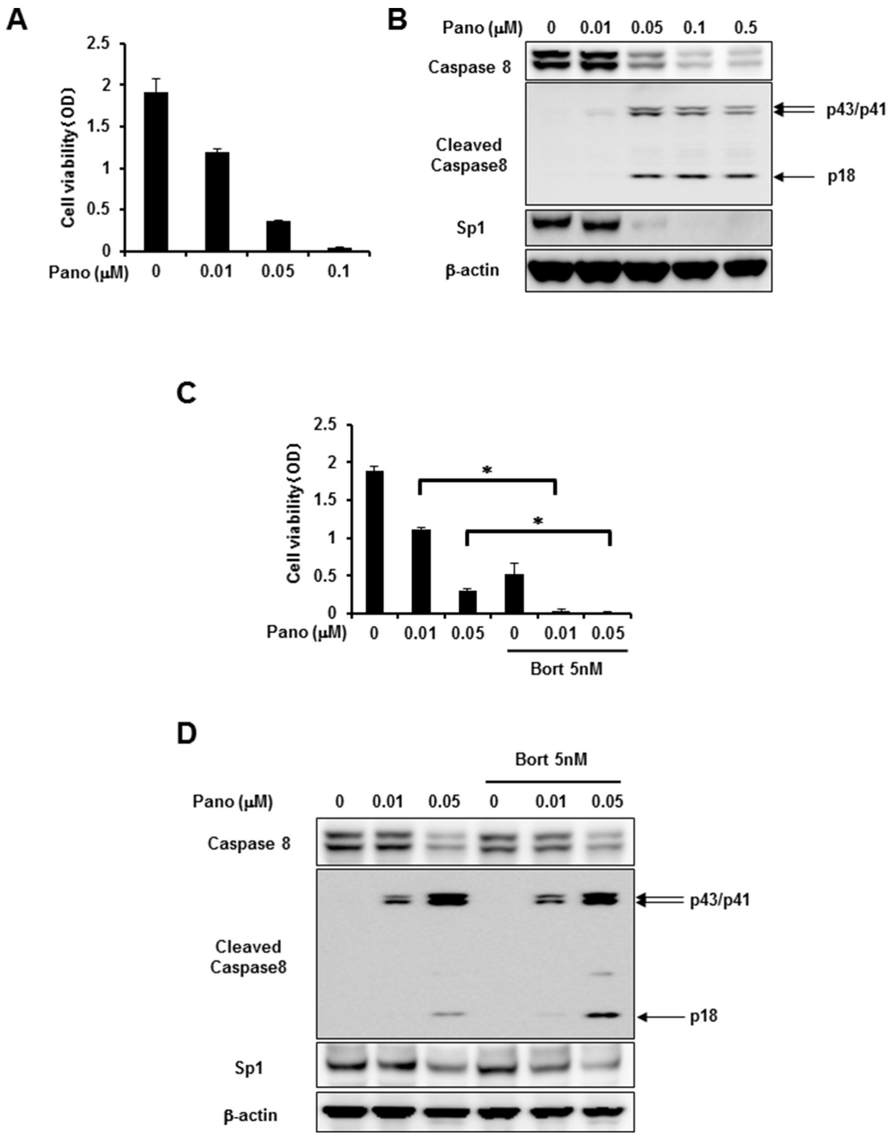

Supplementary Figure S1: Synergistic reduction of MM cell viability and Sp1 protein levels by panobinostat and bortezomib. A. MM cells isolated from a patient with MM were cultured in triplicate in the absence or presence of panobinostat at the indicated concentrations. After culturing for 48 hours, cell viability was measured by a WST-8 cell proliferation assay. Results were expressed as the mean +/- SD. **B.** The primary MM cells were cultured for 24 hours in the absence or presence of panobinostat at the indicated concentrations. Then, the protein levels of caspase-8, cleaved caspase-8 and Sp1 were analyzed by Western blotting. **C.** The primary MM cells were cultured in triplicate in the absence or presence of panobinostat at the indicated concentrations. Bortezomib was added at 5 nM as indicated. After culturing for 48 hours, cell viability was measured. Results were expressed as the mean +/- SD. *p, <0.05. **D.** The primary MM cells were cultured for 24 hours in the absence or presence of panobinostat at the indicated concentrations. Bortezomib was concomitantly added as indicated. Then, the protein levels of caspase-8, cleaved caspase-8 and Sp1 were analyzed by Western blotting. β-actin was used as a protein loading control. Pano, panobinostat; Bort, bortezomib.
